# Examining post-error performance in a complex multitasking environment

**DOI:** 10.1186/s41235-023-00512-y

**Published:** 2023-10-20

**Authors:** Christina M. Lewis, Robert S. Gutzwiller

**Affiliations:** Human Systems Engineering Department, Arizona State University – Polytechnic, SANCA 7271 E Sonoran Arroyo Mall, Mesa, AZ 85212 USA

**Keywords:** Human error, Multitasking, Error-monitoring, Cognitive tunneling, Attention

## Abstract

Previous work on indices of error-monitoring strongly supports that errors are distracting and can deplete attentional resources. In this study, we use an ecologically valid multitasking paradigm to test post-error behavior. It was predicted that after failing an initial task, a subject re-presented with that task in conflict with another competing simultaneous task, would more likely miss their response opportunity for the competing task and stay ‘tunneled’ on the initially errored task. Additionally, we predicted that an error’s effect on attention would dissipate after several seconds, making error cascades less likely when subsequent conflict tasks are delayed. A multi-attribute task battery was used to present tasks and collect measures of both post-error and post-correct performance. Results supported both predictions: post-error accuracy on the competing task was lower compared to post-correct accuracy, and error-proportions were higher at shorter delays, dissipating over time. An exploratory analysis also demonstrated that following errors (as opposed to post-correct trials), participants clicked more on the task panel of the initial error regardless of delay; this continued task-engagement provides preliminary support for errors leading to a cognitive tunneling effect.

Human error is a pervasive issue that has been studied extensively in multiple domains. Real-world work environments such as aviation, air-traffic control, surgery, and military operations are often highly susceptible to human error because of the overloaded and complex nature of the tasks involved (Loukopoulos et al., 2009; Wiegmann & Shappell, 1997). Operators are much more likely to commit erroneous actions when facing an overloaded multitasking scenario (Reason, 1995; Wickens et al., 2022). In aviation, pilots may overly fixate on one task and, unable to efficiently task switch, neglect other important concurrent tasks. Such phenomena, sometimes termed ‘cognitive tunneling,’ result in failure to notice even salient system alarms or attend to altitude maintenance, which lead to catastrophic accidents (Shappell & Wiegmann, 2003; Wickens, 2005). For example, in the real-world scenario of Eastern Airlines Flight 401 (NSTB, 1973), pilots failed multiple attempts to fix a faulty display light, but furthermore they became overly fixated on the mishap. This fixation deterred their attention away from a priority display indicating a status change to a turned-off auto-pilot, leading to a descent for which this neglect eventually led to a fatal crash.

Research has made great strides in producing error classification and cognitive failure models toward predicting error probabilities (Rasmussen, 1983; Reason, 1990; Rouse & Rouse, 1983). These models reliably predict that task demands, which deplete attentional resources, are likely to result in errors in decision making. The research generally relies on cumulative findings from retrospective accident investigation that cannot include experimenter-controlled testing (Dekker, 2002; Shappell et al., 2007), which therefore does not often include measures for specific cognitive mechanisms recruited in these demanding operator scenarios, or measures of any immediate cognitive consequences of errors.

In contrast to the accident-investigation approach, cognitive science research collects direct measurements of cognitive mechanisms. Such research has revealed robust evidence for a neural error-monitoring system which is recruited after a person makes an error (Botvinick et al., 2001; Yeung et al., 2004). Additionally, recent research argues that attention may be maladaptively reoriented after an error (Wessel, 2018). Most research in this area uses controlled, single speeded-choice reaction tasks, such as Flanker tasks and Stroop tasks. The cognitive science approach so far, while investigating mechanisms, does not reflect the task demands of real-world work environments (Wessel, 2012). This represents a disconnect between the highly controlled science of error-monitoring in the domain of experimental psychology, and the application of these findings to complex, high-workload environments where operators commit errors in the field (Wiegmann & Shappell, 2001, 2003).

Given the above, what is the nature of post-error behavior in a realistic high load multitasking scenario? We review the converging evidence for a cognitive mechanism that orients attention toward an error itself immediately after error-commission. We then propose that in a multitasking environment, such a mechanism could be maladaptive as it pulls attention away from other priority tasks, and ironically, such attentional misallocation would then increase the likelihood of an immediate, subsequent error (due to the distraction). Put more simply, committing an error may immediately cause more subsequent errors under these conditions. To test this hypothesis, we conduct a controlled complex multitasking study where participant errors are induced and studied in a novel way.

## Background

### The cognitive error-monitoring system

While the scope of the current paper is focused on a behavioral study, the existing literature consisting of neural measurements provides great insight into post-error effects on attention and action, and so requires a brief review. Specifically, the existence of an error-monitoring system has been supported through neuroscientific data collection, particularly electroencephalography (EEG). EEG studies have provided consistent indices (marked by event-related potentials (ERPs)) of the error-monitoring system, each pertaining to a specific mechanism. One index is the error-related negativity (ERN), a negative wave deflection and reliable component that occurs within 100 ms after incorrect responses in time-pressured choice reaction tasks (i.e., Flanker, Simon and Stroop tasks) (Debener et al., 2005; Falkenstein et al., 1991; Gehring et al., 1993; Holroyd & Coles, 2002; Yeung et al., 2004). A second index is error-positivity (Pe), a positive wave deflection that follows an incorrect response between 200 and 400 ms after the error (Steinhauser & Yeung, 2010; Ullsperger et al., 2010; Yeung & Summerfield, 2012).

Some of the literature suggests that these indexes implicate several important processes that our brains go through right after we commit an error. First, the early wave-deflection is consistently elicited post-error and is suggested to be a preconscious mechanism of error-detection (Botvinick et al., 2001; Holroyd & Coles, 2002; Ridderinkhof et al., 2004; Yeung et al., 2004). Second, with this mechanism elicited, it likely signals the succeeding positive wave deflection, allowing for post-error adjustment (particularly corrective behavior) to follow (Friedman et al., 2001; Nieuwenhuis et al., 2001a, 2001b; Overbeek et al., 2005; Ridderinkhof et al., 2009; Steinhauser & Yeung, 2010). Indeed, there are instances where we see a correlation with the amplitude of both the ERN and Pe with the magnitude of remedial behaviors (such as increased post-error accuracy and post-error slowing, discussed below) (Gehring et al., 1993). Furthermore, we do not usually see the Pe and subsequent corrective behaviors unless there is a preceding ERN index (Nieuwenhuis et al., 2001a, 2001b; Wessel et al., 2011). Therefore, for both mechanisms of the error-monitoring system to be recruited, it is highly likely that there must be error *awareness.* Lastly, research has also shown that the positive wave component of post-error trials has included a key index, the P300 (Arbel & Donchin, 2009). The P300 is notable because it is an index reflecting the recruitment of attentional resources, particularly to deviant stimuli (Donchin & Coles, 1988; Polich, 1986; Rosenfeld & Skogsberg, 2006; Spencer et al., 2001). This is especially amplified in test conditions where accuracy is the participant’s primary priority.

Overall, the neuroscience provides two main implications: first, when avoiding errors is relevant to task goals, the recruitment of the post-error response mechanism is accompanied by an attentional reallocation (reflected by the P300). Second, the initiation of the error-monitoring system is dependent on immediate recognition of the error (reflected by the need for key neural indices to be elicited in order to see the subsequent, corresponding behavioral adjustments). Combined, the neural evidence suggests that the error-monitoring system has mechanisms for error-detection and post-error remedial action, and most notably, that it recruits attentional resources toward an error if detected. Next, we discuss convergent evidence of attention orientation from post-error behavioral measurements.

### Post-error behavior

In speeded forced-choice reaction tasks, when participants commit an error, there have been several consistently observed behavioral adjustments that up until recently, presented as adaptive. First is the slowing of reaction time on trials immediately after error-commission, known as “post-error slowing” (PES). Second, participants tend to (but not always) automatically initiate a correct response on the following trial after an error (Danielmeier & Ullsperger, 2011; Hajcak et al., 2003); post-error slowing allows the participant to execute this remedial action (Rabbitt & Rodgers, 1977). Many scientists regard this control as an adaptive feature of cognition in which focal attention is increased to prevent future errors (Botvinick et al., 2001; Dutilh et al., 2012; Iannaccone et al., 2015; Yeung & Cohen, 2006).

However, opposing theories have argued that the post-error adjustment is instead *mal*adaptive where such processing inhibits ongoing cognition (Wessel, 2018). The maladaptive theory is based off findings that error-commission results in slowing only when errors occur infrequently in choice reaction tasks. If difficulty is increased for a task to the point that errors become frequent, PES no longer occurs; instead, the slowing occurs after the now infrequent correct trials (Castellar et al., 2010; Houtman & Notebaert, 2013; Notebaert et al., 2009). In these studies, it is the novelty of the performance outcome (whether an error or not) that triggers an adjustment mechanism. These data align with the theory that adjustment is an orienting response, in which novelty captures attention (Wessel, 2012), and are further supported by peak amplitude in P300 deflection (a signal of recruitment of mental resources) following novel or surprising events (Friedman et al., 2001). Maladaptivity goes further however and has also shown up in studies with infrequent errors. In studies with very short inter-trial-intervals which obstruct the opportunity for the full cognitive adjustment process, PES even accompanied a *decrease* in post-error accuracy (Jentzsch & Dudschig, 2009). Together, it suggests that post-error adjustment was actually not adaptive (did not result in remedial post-error behavior) when laboratory tasks were altered beyond the traditional parameters of controlled, repetitive tasks such as Flanker, Stroop, or Simon. Additionally, we still see the failed post-error remediation where errors were both infrequent and induced by time-pressured tasks, which are two properties one may expect in more realistic environments. This has an implication for real-world errors, where we might predict operators to react maladaptively to their own sudden errors in high workload scenarios.

### Post-error behavior in multitasking

A significant issue in scaling error-commission findings from research to the real world is that, in many operations, workers are engaged in multitasking (Iqbal & Horvitz, 2007; Loukopoulos et al., 2009; Strayer & Cooper, 2015). In recent years, few researchers have examined how the components of the error-monitoring system (detection and adjustment) might deplete cognitive resources in multitasking scenarios. Steinhauser et al. (2017) used a psychological refractory period paradigm to test how post-error effects differ with varying stimulus onset asynchrony (SOA). Task 1 (a visual flanker task) was followed by Task 2 (an auditory discrimination task) with different SOA times, and the study revealed several relevant findings. Errors on Task 1 led to slowing on both Task 2 and then Task 1 of the next trial, and post-error accuracy increased suggesting a helpful adaptive effect. With increasing SOA, the PES effect on Task 2 disappeared, but still remained for the next trial of Task 1. However, when tasks were more concurrently presented at short SOAs, errors in Task 1 exerted an interfering effect on Task 2 (as PES did not result in increased accuracy). Yet, adaptive PES for Task 1 remained, demonstrating that post-error adjustments can be both adaptive and maladaptive within a multitasking scenario across differing task onsets, and specific to each task. A salient error recruits the error-monitoring system to employ remedial action on the error-specific task, but in turn this pulls attentional resources away from the other task (Schuch et al., 2019). There is also recent (but limited) analysis of ERP components in dual-task performance that reveal diminished activity of the positive wave deflection that indexes task-related attention, corresponding with a reduction in post-error accuracy (Buzzell et al., 2017).

In contrast, Forster and Cho (2014) examined a dual-task scenario with continuous switching between a Stroop and a Simon task, but found post-error slowing was correlated with improved performance on both tasks; the authors argued that the control-adjustment system that is recruited by error-commission had a general adaptive benefit.

There are few studies in the literature that examine post-error accuracy effects in such multitasking conditions, and the findings are conflicting suggesting that more assessment is needed to determine the attentional effects of error-commission. Additionally, the literature appears limited to dual-tasks with simple subtasks using basic stimuli, compared to more applied task-management paradigms with more complex tasks, and with greater numbers of tasks (Wickens et al., 2015).

### Error management frameworks

The role of attention in error-commission has also been examined elsewhere in the field of human factors, with the basic consensus that increasing task demands deplete attentional resources and therefore increases the likelihood of error. Higher workload requires increased focus or attentional allocation, and since resources of attentional allocation are limited in capacity (Wickens, 2002; Wickens et al., 2022), depletion of these resources reduces the ability to handle additional tasks, or unexpected events (i.e., unique problem-solving tasks that are highly dependent on the knowledge-based operations previously outlined by Rasmussen (1983) and Reason (1990)) and increases the likelihood of errors (Gentili et al., 2014; Raby & Wickens, 1994; Wickens et al., 1983, 2022).

A leading theory behind the idea of limited resources in multitasking is the concept of ‘threaded cognition’ (Salvucci & Taatgen, 2008). In this theory-based computational model, cognitive “threads” partially represent multiple resource elements that a task may demand. For example, in aviation, when a pilot must manage multiple tasks, one task is altitude maintenance, which demands a visual processing thread (e.g., processing both the outside environment and relevant information display), in addition to resources needed to enable comparing altitude information against pre-existing knowledge of safe altitude. At the same time, a separate auditory communications task can occur, and its demands will partially overlap (e.g., it will need to use the cognitive ‘threads’ for storing and then comparing verbal information against the relevant visual information on a separate display). In Threaded Cognition terms then, multiple threads can be active in parallel, but only one executive/management resource can be used at a time. In most real-world multitasking, resources tend to be allocated sequentially, where success is highly dependent on cognitive control, and good ‘multitasking’ is actually efficient and continuous task-switching. Such task management becomes increasingly more difficult as workload increases, and subsequently vulnerability to error increases as well (Loukopoulos et al., 2009).

The research examining real-world overloaded task-management scenarios has allowed us to classify the types of errors we can expect, but not the immediate behavioral consequences in the moment of the error. However, one behavioral ‘in-the-moment’ phenomenon is a tendency known in the human factors literature as *cognitive tunneling.* This behavior is defined by a prolonged engagement with a single task, where the operator’s attention is fixated, perhaps on a particular display, leading them to neglect other priority tasks in the working environment (Wickens, 2005). Failure to disengage from this ongoing task and instead ‘tunneling’ into it means that other tasks which require attention may go unnoticed or will not be completed. This phenomenon has real-world implications. For example, in the controlled-flight-into-terrain crash by an Air Force pilot in 2007, the accident report attributed the mishap to the pilot’s ‘target fixation’ on enemy vehicles, while neglecting the other priority task of altitude monitoring (USAF, 2007). Additionally, tunneling itself can be viewed as an error or mistake, given the training and knowledge-based awareness (Bishara & Funk, 2002; Raby & Wickens, 1994) that experienced professionals bring to their jobs. Note, this is different than a performance error, where the operator is unsuccessful in completing a task for which they *are* attending and which is the basis for so much of the research literature in this area.

In this study, we therefore examine whether recognition of error-commission (for performance errors) may lead to such a tunneling effect—that errors recruit attentional resources toward not just the error, but subsequently the error-inducing task or display area. We propose tunneling in general is produced by the following sequence of events: (1) attending to a task, (2) failing to complete it, (3) noticing this failure, (4) focusing on the failure and therefore not attending to other tasks, continuing to respond to the original task. The current experimental paradigm provides measures to test whether this sequence of events occurs in a complex high load scenario and the subsequent outcomes.

## Current study

Based on our study of post-error behavior, there is a substantial gap in the ecological validity of standard laboratory tasks that are used to induce error, which presented an impediment to our attempts to scale it to the real world. In operator environments, rarely are errors committed within a single type of single forced-choice RT task like Flanker or Simon tasks. Instead, the tasks of operators such as pilots, military personnel, air-traffic control, and surgical teams create errors more often because of the high workload and multitasking nature of the operator’s duties.

This article presents an initial study examining the immediate post-error behavioral effects in a multitasking environment. We predict that in a multitasking scenario, error-commission on a task should reorient attention toward the error (and consequently, toward the error-inducing task), temporarily attracting attention away from any competing task, and thereby increasing the likelihood of an error on that competing task. In short, we hypothesize that errors beget more errors even in the more applied domain of our study where multitasking occurs. Further, we used this study to explore whether tunneling behaviors were observed under these error conditions by using mouse movement data.

## Methods

### Participants

Subjects were recruited from the Arizona State University (ASU) Schools of Engineering student population. Participants had normal or corrected to normal vision, were fluent in English, and were at least 18 years old.

A power analysis for a repeated-measures analysis of variance required 34 participants to yield 80% power with a 0.25 effect size (*f* in G*Power). Initially, 37 subjects participated in the study. However, four subjects had to be removed from analysis due to data-loss and outlier performance (described in results section), yielding 33 for final evaluation (*N* = 33; mean age = 24.38 ± 3.63 years), which is more than used in similar studies conducting ANOVA for post-error accuracy (between 20 and 32 participants; Buzzell et al., 2017; Jentzsch & Dudschig, 2009).

Each subject was compensated $20 for 1 h and 15 min of total study session time. The study protocol was approved by the ASU Institutional Review Board.

### Materials

The task was presented on a 13-inch MAC laptop, from which participants sat between 22 and 26 inches away. Participants used a Microsoft surface mouse to interact with the task platform.

**Fig. 1:**
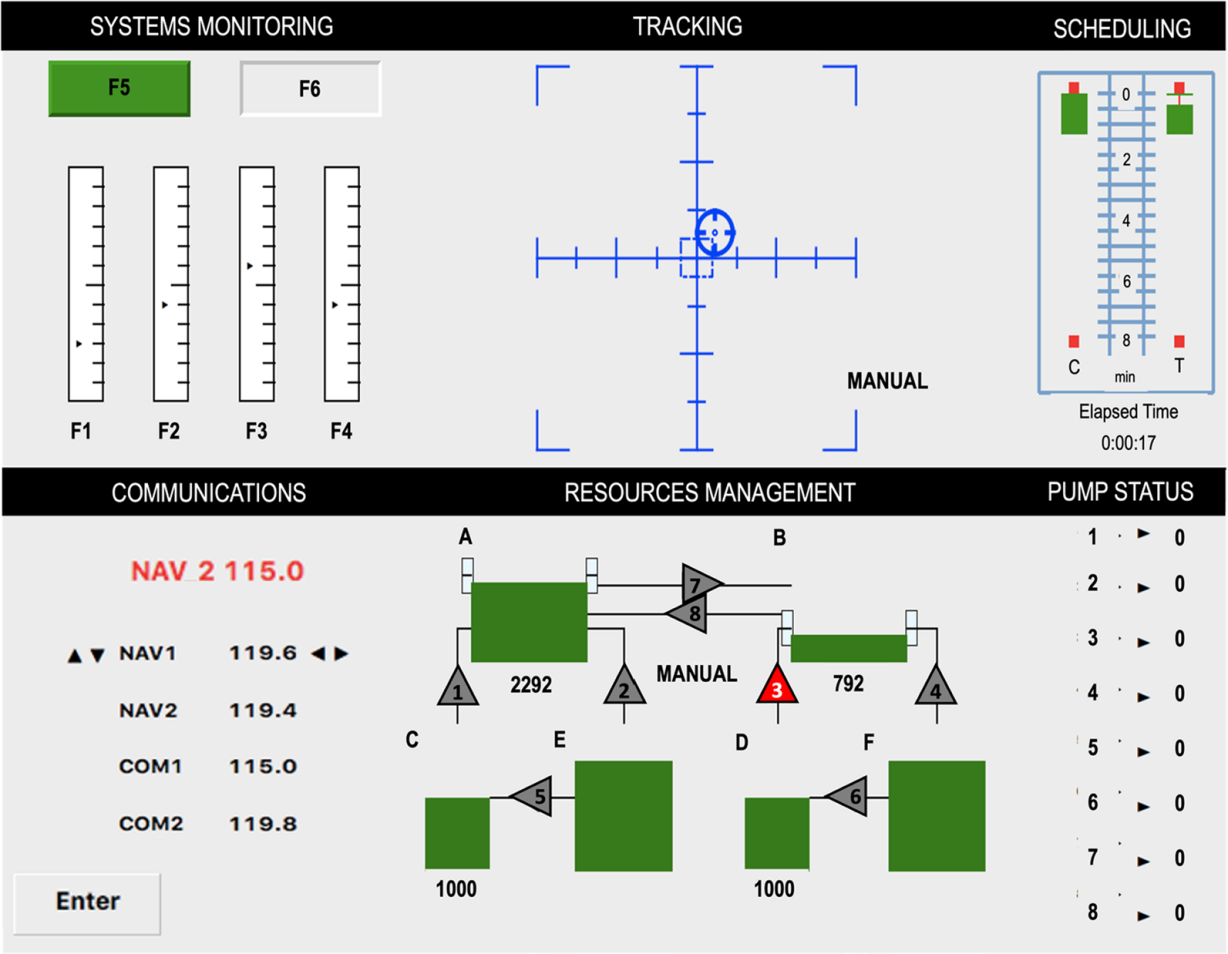
The Open multi-attribute task battery (OpenMATB) Platform—Task Panel Interface. *Note*: Four tasks, clockwise from top left: Systems Monitoring, Tracking, Resource Management, Communications. The Communications (bottom left) and Systems Monitoring (top left; specifically the F5 and F6 alert buttons) are the two critical tasks in this study. The panels on the far right (Scheduling, and Pump Status) are separate activity monitors (not tasks; more details in Table 1)

The Open multi-attribute task battery (OpenMATB; Cegarra et al., 2020) was used to provide the complex task scenario (see Fig. 1). OpenMATB is an open-source platform version of the multi-attribute task battery (MATBII; Comstock & Arnegard, 1992; Santiago-Espada et al., 2011), a research platform developed by NASA to conduct human performance and multitasking workload research. MATB has been used to study the roles of effort, reward, time-on-task, task difficulty, and task priority in an overloading task environment in other multitasking research (Gutzwiller & Sitzman, 2017; Gutzwiller et al., 2016, 2018). The OpenMATB software allows researchers additional access to the backend software coding with highly specific timing of task presentation, salient indicators for task failure, and conflict events using simultaneous task initiation.

A key aspect of the experiment is that error-commission must be detectable (the error event itself must be salient), in order to recruit the cognitive error-monitoring system and orient attention toward the error (Ullsperger et al., 2010; Wessel et al., 2011). Yet, we want the missed opportunity to present ecologically (as opposed to other laboratory tasks, where a missed cue would be followed with something like a red symbol as error feedback). When trying to induce error awareness, the “effector modality” (the physical body part used to give a behavioral response) is a critical element (Ullsperger et al., 2010). For example, in a task that requires a mouse-click, the effector modality is the hand, and actions such as moving the mouse, the click, and the visual presence of the target are all sensory components that serve as evidence of task set implementation. Failure to successfully click the target violates the planned effector behavior (i.e., there is no click, the target is no longer present, the manual plan of action is stunted), which allows awareness of the error-commission. We used constricted manual response, described below, to maximize these effects of error saliency. Lastly, during training, participants are explicitly instructed that the task goal is to respond to their target before it disappears. Therefore, watching the target disappear before you have a chance to click on it is a cue that an error was made—further task parameters are described below.

To induce urgency and competition between multiple alerted tasks, one of the crucial components is that participants are only able to respond with one hand. This ensures they must engage in task-switching to simulate the limitations of operators under high workload, where successful multitasking is dependent on quick and efficient task-switching. All task responses depend on input from a single standard mouse. This also helps meet the requirements for “effector modality” described earlier. Lastly, to remove the confounds of competing modalities in alerting (such as auditory preemption; Wickens et al., 2005) all tasks were solely alerted visually. Figure 1 and Table 1 describe the basic tasks found in OpenMATB: a monitoring task, a tracking task, a resource fuel management task, and a communications task.

**Table 1:** The OpenMATB Platform—task panel configuration

Systems monitoring	Monitoring has two components: 1. Alert lights to be clicked when they change from their base color (if the left green turns gray, or the right gray turns red) 2. Scales with markers continuously sliding up and down, usually within the same horizontal linear space as the other marker. However, when a marker deviates away from this space (sliding to the top or bottom end of its scale), the subject is required to click on that scale to return the marker to its starting center position
Tracking	A blue circular reticle continuously moves with random deviation, farther away from the center crosshair within a square boundary (containing the X and Y axes intersection of the task panel). Participants are required to respond to the task whenever the target reticle moves outside of the boundary, by clicking on the circle and dragging and dropping it back to the center crosshair (the reticle turns green once it is within the center boundary)
Resource management	Participants must maintain fuel levels for two main tanks (A & B), not allowing fuel to go above or below the midline indicators. The flow of fuel to the tanks is controlled through clicking on/off pumps of supply tanks. Certain pumps temporarily fail (lasts 15–30 s) at random intervals, and the participant must work around the failure (e.g., by utilizing other pumps in the system)
Communications	A new radio and frequency configuration will periodically appear red in the top command bar of the communications panel. When this happens, the participant must click on the instructed radio entry field and use increase and decrease buttons (located to the right of the frequency digits) to change the digits to match the red prompt (the digits change at intervals of 0.2, with the total number change being a minimum of 4.0 but maximum of 5.0 unit change). They must then click the “Enter” button to complete the task
Task scheduler	The task-scheduler in the top right corner is not a task. It provides a visual representation of elapsed time for the current session (participants are not required to utilize this scheduler)

The Communications task and the Systems Monitoring task were used for the conflict trials (described below in *Definitions*). To better match the saliency of the conflicting systems monitoring stimulus button, the communications prompt was made the same alert color (same RGB code for red), and the font size of the prompt was enlarged two sizes above the rest of the platforms text (a font size of 17px was the largest to ensure two degrees of visual angle, considering display size and average distance between the display and participant)

#### Definitions

##### Test trial

**Fig. 2:**
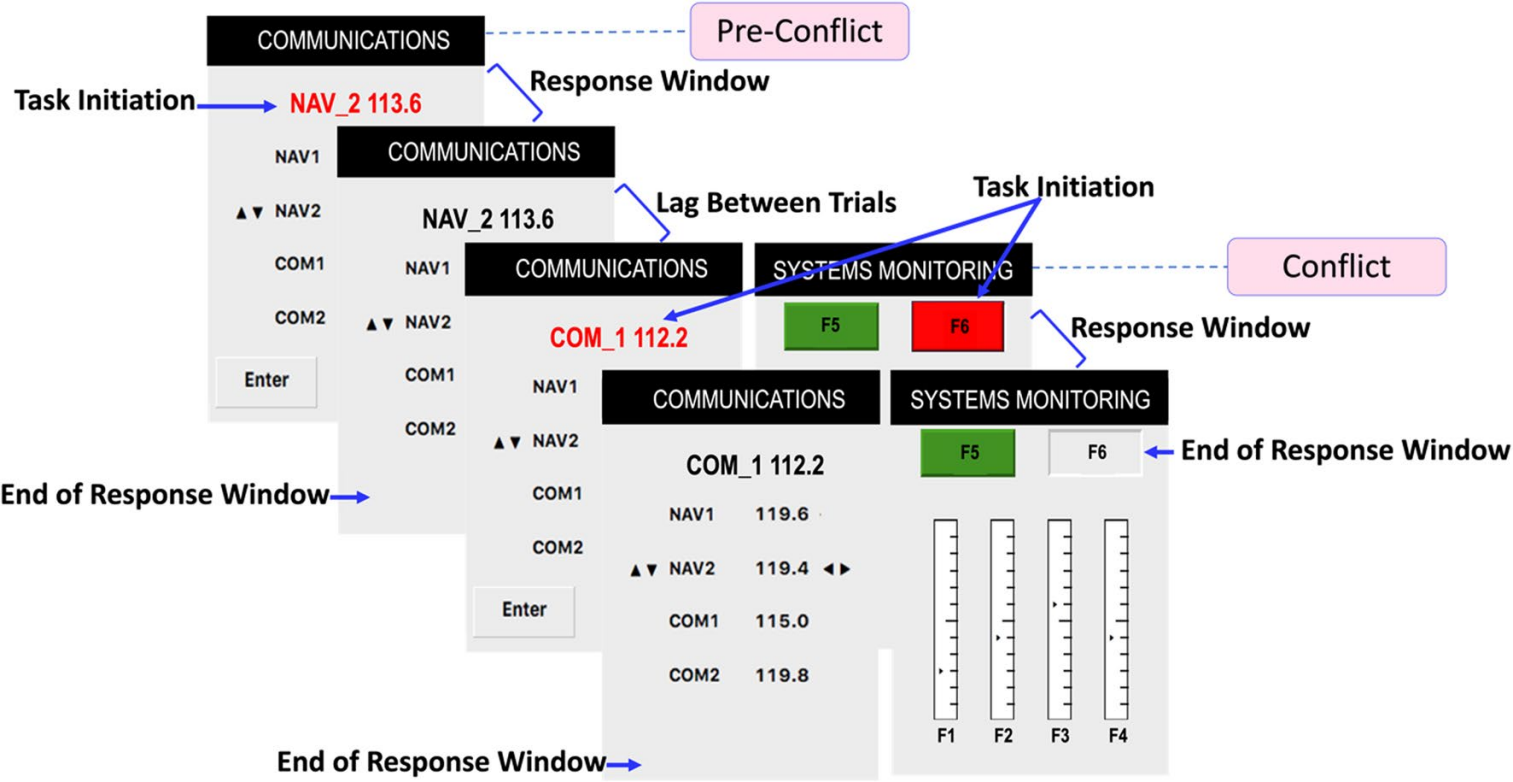
Test Trial Sequence Diagram. *Note*: A trial sequence that starts with an induced-error task (pre-conflict) and is followed by a conflict

The test trials (Fig. 2) involved a sequence of events created to induce an initial error on one task and then examine the subsequent effect of this error when that same task is quickly re-alerted alongside a simultaneous competing task. The sequence starts with (1) the communications task presentation (induced-error task), (2) then a response to the task is captured, (3) a short lag of time passes, and (4) another communications task is presented *again but simultaneously* with the red-light alert of the systems monitoring task. This creates a decision point (i.e., a conflict) in task choice.

##### Performance error

Any failure to complete a task correctly is a performance error. These errors were induced by limiting the window of time that the subject must respond to the task. The response window begins from task-initiation (i.e., the right monitoring button turns red, or, the new radio-frequency units appear red inside the communications command bar), and after a short amount of time the response window ends (i.e., the monitoring button returns to gray, or the "Enter" button disappears and the radio-frequency prompt turns back to black). Both a failure to respond to the task within the allotted time (i.e., a miss), or entering the wrong response, are considered performance errors. (*Note:* the text of the radio-frequency prompt remains the same until the next red alert).

##### Pre-conflict (induced-error task)

The communications task was used to induce the initial performance errors. To produce as close to a 50% error rate as possible, the length of the response window was staircased throughout the experiment for the pre-conflict communications task, meaning that on correct trials (where participants respond in time with the correct radio-frequency entry), the time window for the next pre-conflict communications task would be reduced by 1.5 s. Additionally, keeping in mind that with each interaction a practice effect can decrease the likelihood of error, the time window increased by only 1 s in the case of errors. The response window for the first trial in each of the sessions began at 10 s, a time constraint used in Gutzwiller (2017) to induce high load parameters while keeping response opportunity possible. We targeted a 50% error rate, which would allow for a relatively equal comparison of post-correct and post-error measurements. The staircasing only effected the response window of the initial pre-conflict communications task—as soon as this response window ended (whether shorter or longer due to the staircase iteration), the remainder of the trial sequence (lag condition + conflict) immediately followed.

##### Conflict

All induced-error communications tasks were followed by a conflict (see Fig. 2). A conflict occurs when a communications task and a systems monitoring task are initiated at the same time. A conflict provides the opportunity to determine which task the participant chooses to attend to after they have committed an error. The response window for the monitoring task was 3 s, while the communications task was available for 10 s.

##### Lag

**Fig. 3:**
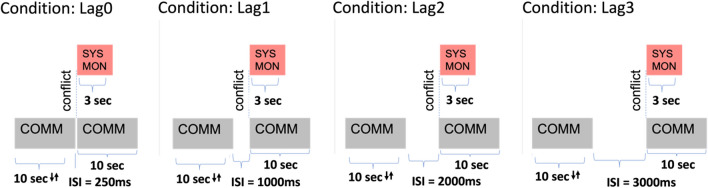
Lag Condition Diagram. *Note*: An induced-error task (communications) followed by a conflict at different lag conditions. Arrows here indicate the varying response time window for the pre-conflict communications task due to staircasing. Regardless of the pre-conflict duration, once the window ends (e.g., the ‘Enter’ button disappears), the lag + conflict sequence immediately follows

Lag is the amount of time between the end of the induced-error task and the initiation of the following conflict being presented. There were four lag conditions: one where the conflict started immediately after the end of a previous communications task (250 ms), and three other lag conditions at 1, 2, and 3 s (see Fig. 3).

This range of lag times was used to induce time-pressured inter-stimulus-intervals (ISIs) at realistic intervals, such that we could test the effect of maladaptive attentional reorientation in conditions of shorter lag times (in part because previous studies with shorter ITIs remove the window of opportunity for adaptive effects; Buzzell et al., 2017; Jentzsch & Dudschig, 2009).

### Procedure

Participants were given 10 min to review training material, which consisted of a slideshow that provided images of the platform and details on how to accurately complete each task. Participants were also given time to ask any clarifying questions on how to respond to each of the four tasks. This was followed by a 2-min practice session so that the subjects could become accustomed to the platform. After practice, participants completed the experimental portion. Participants were instructed that they should try to perform all tasks as quickly and accurately as possible.

For each participant, there were four test sessions, each 10 min long. Each session contained eight periodic test trials of induced-error tasks and conflicts, randomly scheduled to occur within the session. Within a session across the test trials, there were eight lags experienced (two of each lag type) between pre-conflict and conflict tasks, administered in random order. Between test trials, all four tasks in MATB were randomly and periodically initiated to represent a free-choice multitasking environment that requires continuous monitoring by the participant. The between-test trial task schedule for each of the four sessions was based on the following parameters: Systems Monitoring had between 12 and 18 alerts with randomized selection of light alert versus scale alert, and which light number and scale number/direction. Resource Management task had 6–10 pump failures, with randomized pump selection. The Communications task had 3–6 alerts with randomized radio and frequency target selection. The tracking task ran continuously except when periodically timed-out on auto-mode. These numbers are close to the amount of task alerts used in previous studies that aimed to present subjects with a challenging, but manageable, multitasking environment (Gutzwiller & Sitzman, 2017; Wickens et al., 2016).

During a test trial sequence, none of the other tasks alerted (the tracking and resource management tasks were put on automatic solver mode). To prevent this change to auto-mode from leading to a priming effect for the test trial sequence, the auto-change occurrences were set to occur within a random time 1—5 s before a test trial sequence. The auto-modes also occurred periodically an additional 2–6 times, lasting 5 s, in the broader multitasking session *without* leading to a trial sequence. The goal was to have four experimental sessions that were equally challenging but diverse scenarios, and for all subjects to experience the same degree of overloaded task environment. All subjects were presented with the same task schedule.

The pre-conflict communications task should result in performance error for about half the trials, due to staircasing described earlier, which were labeled either *error* or *correct* trials based on participant performance. The primary outcome measure is the performance on the *subsequent* systems monitoring task in the conflict event that follows. The performance measure has two levels: (1) the outcome following an initial pre-conflict error (a *post-error* score) or (2) the outcome after a participant correctly responds to the pre-conflict trial (a *post-correct* score). More specifically, this outcome is calculated using the proportion of systems monitoring errors out of the total conflict trials for each lag condition, which is referred to as the *error-proportion.*

#### Hypotheses

The prediction is that the error-monitoring network activates on participants errors for the pre-conflict communications task; this initial error then shifts attentional allocation causing fixation on their error. In the following conflict then, it is expected that the participant will not have the attentional resources to allocate to the newly presented systems monitoring task—and will be more likely to error on it, which would then result in another, subsequent performance error (this time on the monitoring task). This leads to the first hypothesis:

H1**: **errors on the pre-conflict task, as compared to accurate performance on the pre-conflict task, will lead to significantly more errors on the subsequent conflict trials.

Post-error effects such as PES and increased accuracy are observed in trials that immediately follow errors in studies whose inter-trial intervals (ITIs) ranged from 1–2.5 s (Castellar et al., 2010; Debener et al., 2005; Endrass et al., 2005; Nieuwenhuis et al., 2001a, 2001b), so we assume that the effects last at least as long as the average ITIs in these studies. However, the strength of the effect may vary within that range. For this reason, we explore the error-awareness effect up to 3 s after error-commission; of course, it is possible that the attentional depletion is most disruptive immediately after the error-commission as opposed to a few seconds after. This leads to the second hypothesis:

H2: the highest post-error error-proportions will be when a conflict is initiated in the no-lag condition immediately after (0.25 s) the end of pre-conflict tasking.

## Results

Due to lags in the program’s refresh rate for two subjects, more than three of their 32 test trial task alerts were omitted. These data were removed before data analysis. Additionally, there were two subjects whose count of false entries for the communications task was more than three standard deviations above the mean for all participants. A large amount of false entries (as opposed to missed entries) reflects a lack of diligent attention to the task, or a misunderstanding of the task instructions, which requested responding to both portions of the prompt and entering the correct radio and frequency before submitting a response. Subsequently, these two subjects were also removed. All following analyses are performed on the data from 33 remaining participants.

### General performance

The scope of this manuscript is focused on performance within the conflict trial sequences. However, the overall interaction with all four tasks not including the conflict trial sequences was also analyzed to gauge participant engagement with the entire suite of tasks. For the tracking and resource management task, the count of reticle drags and pump clicks, which were reflective of participant effort to respond to the tasks, was calculated across all trials. All subjects were within two standard deviations from the average of the whole sample. For the communications and systems monitoring task (including all scale and light alerts), the rate of successful responses was calculated across all trials. The average accuracy for systems monitoring was 71.37% (SD = 14.17%), and the average accuracy for communications was 73.03% (SD = 24.35%). Poor performance scores were infrequent, found among three to five subjects for each task, and mostly in the first trial after which performance improved. Overall participants appeared to continuously engage with the dynamic multitasking platform and put in valid effort to respond accurately to all four tasks.

### Staircase test

As a manipulation check, we examined whether the staircased timing on pre-conflict trials did indeed induce enough errors to further examine their effect on performance. The proportion of miss trials (*M* = 0.53, 95% CI [0.50, 0.56]) was only slightly, though statistically significantly higher than 50%, confirmed by t-test (*t*(131) = 2.01, *p* = 0.047). This rate was within reason, allowing for a comparison between the two conditions (post-error versus post-correct), but there are implications for fine-tuning staircasing methods in future studies.

### Post-error versus post-correct performance

A 2 × 4 ANOVA was used to examine the effects of pre-conflict performance (either correct or error), and lag condition (0.25, 1, 2, or 3 s after), on the error-proportion in conflict trials (the amount of misses on the systems monitoring task of the conflict trials). Because Mauchly’s test for sphericity was significant for lag and the interaction term, the *df* and *p*-values reported incorporate Greenhouse–Geisser correction.

**Fig. 4:**
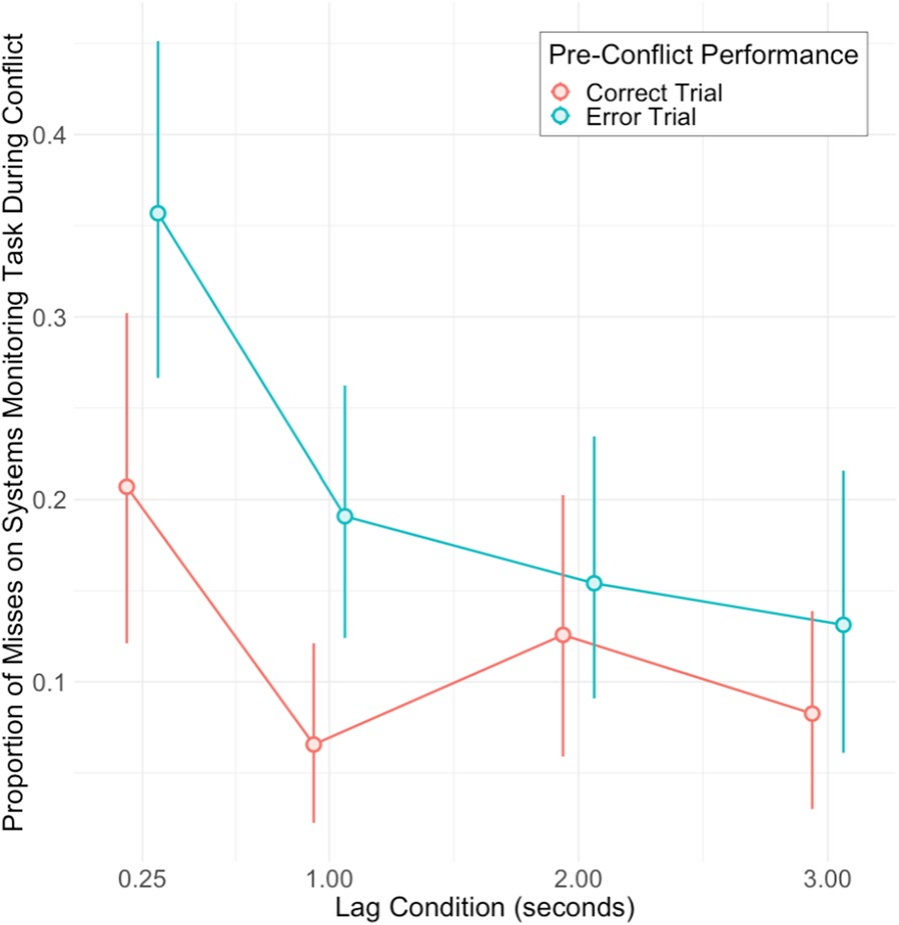
Pre-Conflict Performance Effects on Monitoring Task Accuracy During Conflict. *Note*: Effects of pre-conflict performance (either correct or error communications trial, error trials defined by misses) on the following conflict trial error-proportion (proportion of misses on the systems monitoring task) across four lag conditions. Error bars are bootstrapped 95% CIs (*n* = 33)

Results of the analysis reveal a main effect of pre-conflict performance, where pre-conflict trials with error (a miss on the communications task) led to a significantly higher error-proportion on the systems monitoring task on the following conflict trial [*F*(1, 32) = 13.13, *p* < 0.01, $\eta_{p}^{2}$  = 0.29]. There was a main effect of lag condition where the error-proportion was highest on conflict trials which appeared immediately (0.25 s) after the pre-conflict response and gradually decreased as the lag condition increased [*F*(2.63, 84.14) = 10.93, *p* < 0.001, $\eta_{p}^{2}$  = 0.26]. The interaction term between pre-conflict performance and lag condition was not significant [*F*(1.99, 63.55) = 1.95, *p* = 0.151]. Figure 4 shows the difference between error-proportions following error, versus correct trials, in each of the four lag conditions, and supports our first hypothesis.

While the initial performance error was defined by misses on the pre-conflict communications task, a small portion of performance on the task was made up of false entries (subjects clicked ‘Enter’ on time, but with the incorrect frequency or radio selected). However, only 5.6% of the pre-conflict error trials were defined by these false entries. If these are included in the analysis, the ANOVA results are nearly identical—a main effect of pre-conflict performance [*F*(1, 32) = 11.95, *p* < 0.01, $\eta_{p}^{2}$  = 0.27], a main effect of lag condition [*F*(2.65, 84.89) = 10.91, *p* < 0.001, $\eta_{p}^{2}$  = 0.25], and an insignificant interaction term [*F*(2.05, 65.58) = 1.66, *p* = 0.198]. Given the negligible difference, pre-conflict errors on the communications task will only be defined by misses throughout the article. Additionally, this falls in line with the saliency feature of performance error, where participants must notice the error (being overtly alerted to the missed opportunity when the enter-button disappears) in order to recruit attentional orientation.

In order to examine accuracy between post-error and post-correct trials, post hoc contrasts were conducted at each lag condition (Bonferroni correction was applied for the four tests; Table 2). Significant differences between post-correct and post-error error-proportions, particularly for the shorter lag conditions, were found. Lag conditions of 0.25 s (*M* = 0.36, SD = 0.28) and 1 s (*M* = 0.19, SD = 0.21) for post-error trials had significantly greater error-proportion compared to post-correct trials (*M* = 0.21, SD = 0.26 and *M* = 0.07, SD = 0.15) ([*t*(126) = − 3.43, *p* < 0.01, *d* = 0.31] and [*t*(126) = − 2.86, *p* = 0.02, *d* = 0.26], respectively), while the latter two lag conditions did not show a significant difference. Note, however, that there is still not a significant interaction between the factors. Overall, the significant differences between the immediate lag conditions and the later ones provide partial support for our second hypothesis.

**Table 2:** Correct trials versus error trials by lag condition: Post hoc contrasts on conflict trial miss proportions of systems monitoring task

Lag condition (seconds)	*Estimate*	SE	*df*	*t*	*p*	*d*
0.25	− 0.150	0.044	126	− 3.425	0.003	0.31
1	− 0.125	0.044	126	− 2.864	0.020	0.26
2	− 0.028	0.044	126	− 0.648	1.000	0.06
3	− 0.049	0.044	126	− 1.114	1.000	0.10

*p*-values represent multiple comparisons that were adjusted using Bonferroni correction

### Cognitive tunneling analysis

A sequence of events was described in our background section to explain the possible scenario of error-induced cognitive tunneling. To test whether the events were reflected in the participants' behaviors, an exploratory analysis was conducted to examine the degree of continued interaction on the error-induced task (communications). In the tunneling process, the participant would be focused on their recent error-commission, followed by a focus on the task panel where this error occurred (the communications task initiated during the conflict trials). Continued focus on the communications task could be reflected in engagement with the task panel used for task performance (i.e., clicking on an entry field). Number of mouse-clicks on the communications panel was measured starting from the final response of the pre-conflict trial until the monitoring task alert ended. Conservatively, clicks were not counted after the end of the monitoring task alert, given the expectation that (1) the cognitive tunneling effect tied to error-preoccupation would not last longer than that, and (2) after the monitoring task alert ends, there are no longer two obviously conflicting tasks competing for attention.

A 2 × 4 ANOVA with the pre-conflict performance factor (error versus correct) and the lag condition factor (0.25, 1, 2 or 3 s) was conducted on the dependent variable of average mouse-click count on the communications panel during conflict. Mauchly's test for sphericity was significant for Lag and the interaction term, the *df* and *p*-values reported incorporate Greenhouse–Geisser correction.

**Fig. 5:**
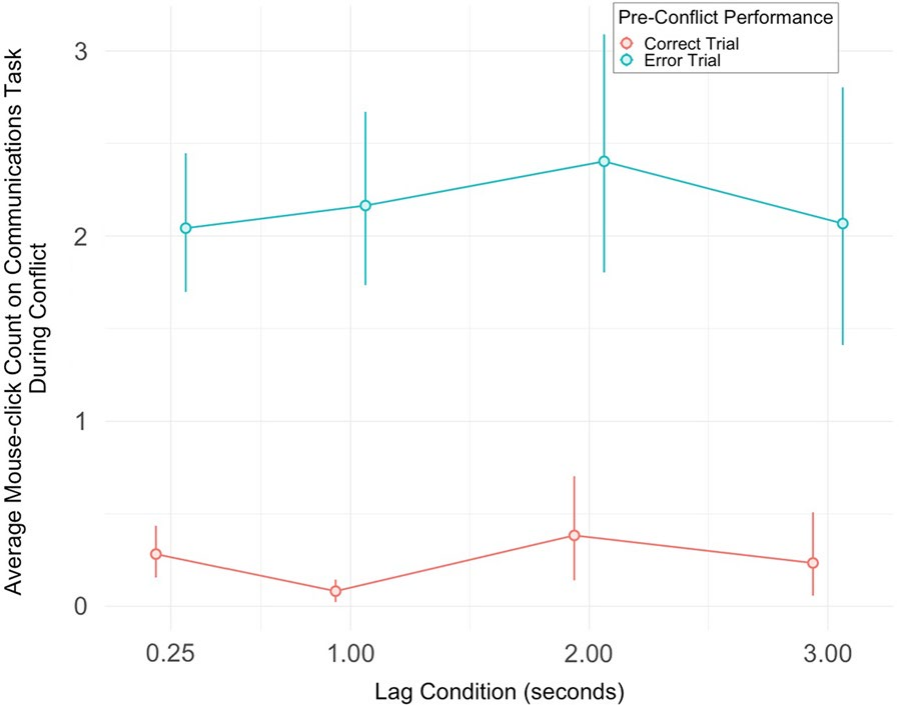
Pre-Trial Performance Effects on Subsequent Task Engagement (Average Mouse Clicks). *Note*: Effects of pre-conflict performance (either correct or error communications trial, error trials defined by misses) on the following conflict trial mouse-click engagement on the communications task, as a function of lag condition. Error bars are bootstrapped 95% CIs (*n* = 33)

Results reveal that errors on pre-conflict trials (a miss on the communications task) resulted in a significantly higher average mouse-click count (post-error: *M* = 2.17, SD = 1.67; post-correct: *M* = 0.25, SD = 0.60) on the communications task within the following conflict trial [*F*(1, 32) = 114.01, *p* < 0.001, $\eta_{p}^{2}$ = 0.78]. There was no effect of lag however [*F*(2.10, 67.20) = 0.65, *p* = 0.535, $\eta_{p}^{2}$  = 0.02], and no interaction [*F*(2.38, 76.13) = 0.39, *p* = 0.714, $\eta_{p}^{2}$  = 0.01]. Figure 5 illustrates the difference between average mouse-click counts on the communications task panel that were preceded by error in the communications task, versus correct trials, across the lag conditions. Following an error, participants were over 8 times more likely to continue clicking on/interacting with the communications panel during the subsequent conflict task interval.

**Fig. 6:**
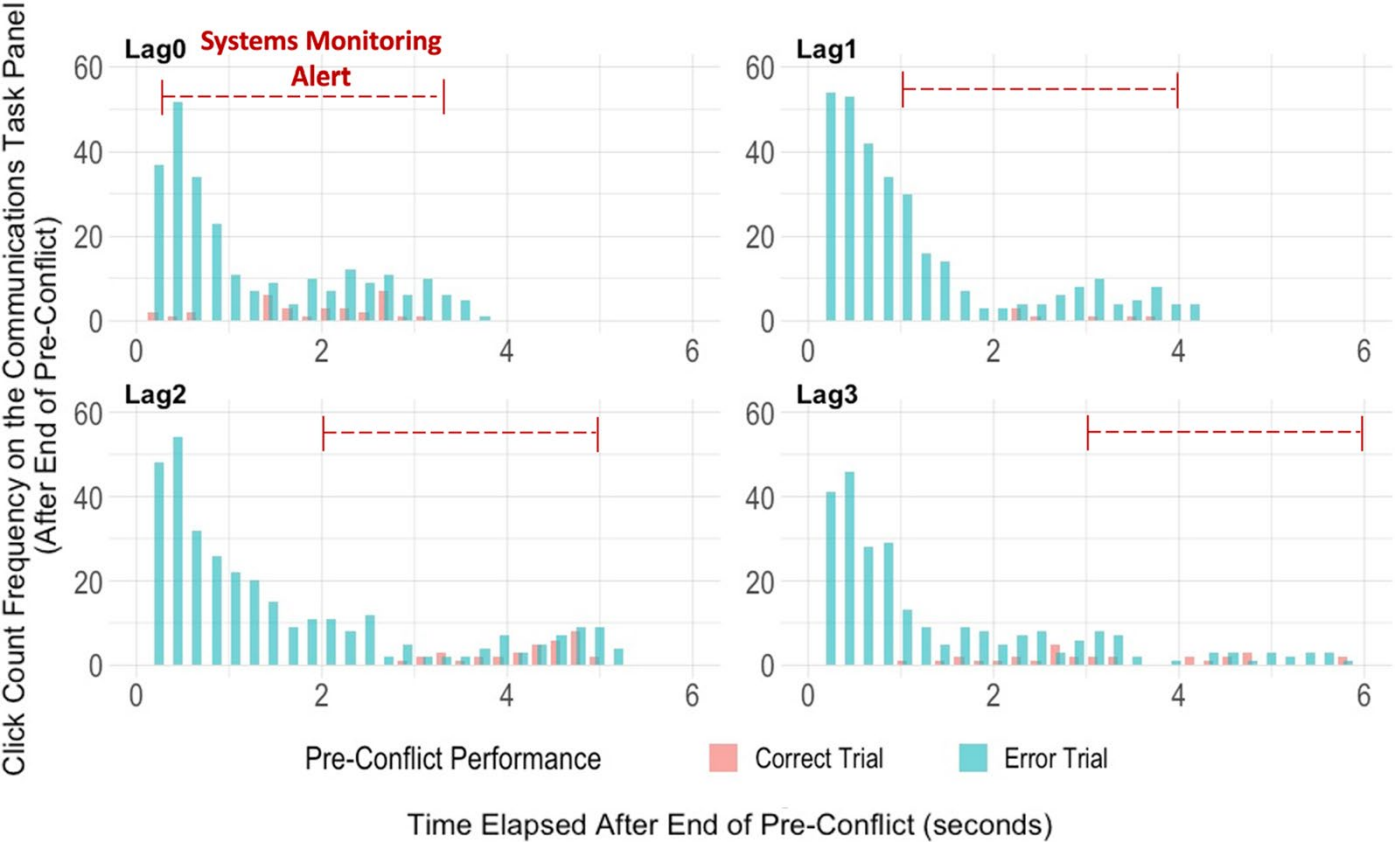
Pre-Trial Performance Effects on Subsequent Task Engagement (Mouse Clicks Over Time). *Note*: There are four graphs, one for each lag condition: Lag0, Lag1, Lag2, and Lag3. For each, the overall click count for participants who clicked on the communications task panel following pre-conflict (*y*-axis), are plotted over time (*x*-axis), which includes the duration of the lag and the subsequent systems monitoring conflict. Additionally, click counts differ by pre-conflict performance

While the average click count gives us some preliminary information on the difference between post-error versus post-correct behavior, understanding the click rate (when within the lag do the clicks occur?) would paint a clearer picture of how a cognitive tunneling effect may be manifesting. Using the same section of the trial sequence (right after the end of the pre-conflict, up until the end of the systems monitoring alert during the conflict), we counted frequency of clicks as they occurred over time. Figure 6 demonstrates a higher post-error click count (as compared to post-correct) for all four lag conditions.

Interestingly however, a large portion of these clicks occur within the first two seconds following the end of the pre-conflict window. The click rate suggests a rushed clicking behavior on the errored task panel immediately following an error. On the trials where the lag condition was shorter (0.25 or 1 s), this further explains how participants are more likely to miss the systems monitoring alert (post-error) during conflict. One interpretation is that this is a representation of error-induced cognitive tunneling on the communications task, demonstrated by a fixated continued physical engagement; so that participants are attending to (and physically engaged with) the communications task panel during the critical time when the systems monitoring alert appears for a limited duration.

Furthermore, a Pearson correlation run across all trials shows a significant relationship between the average click count on the communications task panel (Fig. 5) and the average error-proportion (misses; Fig. 4) on the systems monitoring task during conflict [*r*(262) = 0.30, *p* < 0.001]. Additionally, this click average is also correlated with the reaction times on the systems monitoring task trials that had successful responses [*r*(256) = 0.27, *p* < 0.001] (reaction time calculated from the start of the systems monitoring alert until a successful click response on the alert). At the end of the pre-conflict window, when there is more clicking behavior on the communications task panel, it is more likely that the participants will either take longer to respond to the systems monitoring alert during conflict, or entirely miss the alert altogether.

Lastly, when comparing the reaction times on successful systems monitoring responses during conflict, the overall average is slightly higher post-error (*M* = 1.44 s, SD = 0.44), versus post-correct (*M* = 1.34 s, SD = 0.31). While the difference is significant [*t*(228.55) = − 2.00, *p* = 0.049], this is only due to the significant difference at the shortest lag condition (post-error: *M* = 1.74 s, SD = 0.55, versus post-correct: *M* = 1.36 s, SD = 0.28)[*t*(47.29) = − 3.34, *p* < 0.01], with no significant difference at the other three lag conditions. One interpretation for these findings is that the post-error decrement (presented as either an entire miss on the systems monitoring, or a longer reaction time on the task if successful) is largely due to physical preoccupation with the communications task panel (a high number of rushed clicks) during the first 1 s after the end of pre-conflict. On the shortest lag condition, this would cut into the critical response period for the subsequent systems monitoring task.

As mentioned in the background research, part of the function of the cognitive error-monitoring system is to be remedial. In single forced-choice reaction tasks, this means improved accuracy on that same task following an error. While we did not have a hypothesis about what this would mean for an errored-task in a multitasking scenario, we conducted an exploratory analysis to compare performance on the communications task pre-conflict and during conflict. If focus is reoriented on the communications task-panel after making an error on it, it makes sense that increased engagement would result in higher chance of accuracy. Indeed, we found that the overall rate of misses on the communications task during conflict following a miss on the task (*M* = 0.36, SD = 0.28) was lower compared to the pre-conflict miss rate (*M* = 0.53, SD = 0.18). A pairwise contrasts analysis revealed that this difference was significant at lag conditions of 1 and 2 seconds ([*t*(113) = − 4.52, *p* < .001, *d* = 0.42] and [*t*(113) = − 3.71, *p* < 0.01, *d* = 0.35], respectively), but not 0.25 and 3 seconds. The results are interesting, where the error on a specific task would not immediately result in improved focus on that task when it re-occurs 0.25 s later, but soon after it would be remedial to that task. In fact, within the first second the error-induced distraction is potentially detrimental to *both* competing tasks in a multitasking scenario.

## Discussion

The results from the analysis comparing post-error accuracy against post-correct accuracy supported both hypotheses 1 and 2. Overall, error-proportion on the competing task was significantly higher after initial error-commission, and we saw that this error-proportion appears highest on conflict trials that occurred immediately after the error (though the overall interaction effect was non-significant). If committing an error causes maladaptive attentional reorientation, this effect dissipates over time (over 3 s); there should be an interaction. One possible explanation is that the experimental paradigm did not provide enough test trial sequences (32 test trials, with 8 data points for each lag condition, which may not be adequate to power an interaction effect). Of course, one of the challenges in this paradigm is inducing enough errors to properly contrast error and correct performance effects. This part of the paradigm was less ecological, as real-world errors would occur much less frequently than a rate of 50%, yet we needed an initial measure to comparatively examine post-correct versus post-error behavior—future study iterations will include self-induced error-rates. Here, the staircase timing yielded a slightly more error-prone pre-conflict performance (~ 53% miss rate); traditionally, the use of staircased response windows occurs over far more repeated trials in order to eventually yield the 50% performance; in future replication efforts, increasing the number of test trials could even this out.

Regarding cognitive tunneling, the mouse-click data proved to be very promising. The data strongly suggested that errors result in continued interaction with the errored task when it is presented again and in conflict with another equally important task to do. The continuous engagement appears to be an error-induced cognitive tunneling phenomena, which mimics that seen in more complex domains, such as the case of pilots becoming engrossed with a task or mishap and ending up in a descent into the ground due to the preoccupation (e.g., Eastern Airlines Flight 401, or United States Air Force 524th Expeditionary Fighter Squadron; NTSB, 1973; USAF, 2007). Inducing these types of behavior has traditionally been quite difficult to do in applied settings with only a handful of experimental reports available (Dehais et al., 2014; Wickens, 2005). This beckons further analysis and also suggests one of our major novel contributions is from using work from basic research and applied cognition to create a paradigm to induce the tunneling effects.

Additionally, it is important to address the necessity for error-recognition, for which failure to complete a task must be salient (noticeable). Without awareness of a failure, consequences may not be attributable to the proposed error-cascading effect. While this study was still very controlled, we chose to be more ecological in the paradigm with (1) the types of tasks involved (e.g., a 2D representation of some of the tasks involved in a flying a plane) and (2) the way an error would be detected—through the stunting of an effector action (missing the click of the enter-button, particularly in that it was preceded with clicks on the other entry fields of that task, showing attempted action). However, future directions could utilize EEG measurements to ensure that additional indexes of error-detection (i.e., the ERN) accompany the subsequent error-cascading while participants interact with the multitasking platform. Additionally, using eye-tracking could provide additional evidence for post-error cognitive tunneling. This would be useful in determining whether visual fixation occurs on the task panel on which a participant just committed an error, and would also make clear what information is available in the subject’s visual field at the time. Discerning whether competing alert information is encompassed within the subject’s spatial attention while fixated on the errored-task panel would help portray the degree of error-induced cognitive tunneling (i.e., competing alerts could be fully perceivable, and yet still neglected).

Lastly, this study used two specific tasks for the conflict pairing—communications and systems monitoring. The other two tasks are continuous in nature. How attention is recruited for a continuous task, versus toward an abrupt alert, could have posed confounds for post-error results. These differences were considered when designing for the goal of this study, which was to first establish the post-error decrement effect, include ecologically valid elements in the paradigm, and control for some confounds these elements present. This included factors like: (1) programming the abrupt alerts of tasks according to the exact timing of the conflict sequence schedule, (2) only starting with one presented task (communications) in the sequence to control when and how the initial error occurs, and (3) matching the RGB color code of the alert signals. Now that we have observed the post-error effect using the controlled measures, future iterations of this study can include different task pairings, along with other alert modalities (i.e., auditory alert on communications), apparatuses (i.e., joystick apparatus for the tracking task), and more dynamic timing (i.e., the initially errored-tasks can occur while another task has already been alerted).

### General discussion

The aim of this article was to explore how errors induce a distraction away from competing tasks in a multitasking scenario, resulting in higher likelihood of missed performance on those tasks. We use a more ecologically valid overload scenario as compared to the tasks used in basic research, in which we could still induce tightly controlled error in participant responses. Post-error research in the fields of cognitive and neuroscience has presented empirical data supporting the concept of a neural mechanism that recruits cognitive resources to implement post-error adjustments (Danielmeier & Ullsperger, 2011; Wessel, 2012). The evidence suggests that the adjustment mechanism depletes attentional resources. However, attention is needed for successful performance in complex and demanding scenarios. Therefore, it stands to reason that when a subject commits an error in a multitasking environment, subsequent error becomes more likely (given the loss of attentional resource that could otherwise be used to prevent future errors)—the ‘error cascade’. Indeed, this is what was found in the current study, and although errors are typically rare (the very thing that makes them distractions), establishing behavioral indices of error-cascading behavior in operators should elicit potential insights into error recovery.

#### Open practices statement

This study is not a pre-registered report. Data are available via the Open Science Framework here: https://osf.io/audkj/?view_only=b3654cd708bc44acb130004259d34904. Additional information is available upon request to the corresponding authors.

## Data Availability

The final datasets used for analysis are available via the Open Science Framework under: https://osf.io/audkj/?view_only=b3654cd708bc44acb130004259d34904. Analysis scripts are available upon reasonable request to the corresponding authors.

## Abbreviations

EEG: Electroencephalography
ERP: Event-related (brain) potential
ERN: Error-related negativity
ITI: Inter-trial interval
MATB: Multi-attribute task battery
PES: Post-error slowing
SOA: Stimulus onset asynchrony
